# Development and Evaluation of Jaw Position Detection Method in Jaw-tracking Delivery Using an Electronic Portal Imaging Device Cine Mode

**DOI:** 10.14789/jmj.JMJ22-0014-OA

**Published:** 2022-11-18

**Authors:** TORU KAWABATA, SATORU SUGIMOTO, CHIE KUROKAWA, KEISUKE USUI, TATSUYA INOUE, HIRONORI NAGATA, HIROYUKI WATANABE, KEISUKE SASAI

**Affiliations:** 1 Department of Radiation Oncology, Graduate School of Medicine, Juntendo University, Tokyo, Japan; 1 Department of Radiation Oncology, Graduate School of Medicine, Juntendo University, Tokyo, Japan; 2 Varian Medical Systems, Tokyo, Japan; 2 Varian Medical Systems, Tokyo, Japan; 3 Department of Radiation Oncology, Faculty of Medicine, Juntendo University, Tokyo, Japan; 3 Department of Radiation Oncology, Faculty of Medicine, Juntendo University, Tokyo, Japan; 4 Department of Radiological Technology, Faculty of Health Science, Juntendo University, Tokyo, Japan; 4 Department of Radiological Technology, Faculty of Health Science, Juntendo University, Tokyo, Japan; 5 Department of Radiation Oncology, Shonan Kamakura General Hospital, Kanagawa, Japan; 5 Department of Radiation Oncology, Shonan Kamakura General Hospital, Kanagawa, Japan; 6 Graduate School of Health Sciences, Showa University, Kanagawa, Japan; 6 Graduate School of Health Sciences, Showa University, Kanagawa, Japan

**Keywords:** radiotherapy, quality assurance, jaw-tracking technique, multileaf collimator

## Abstract

**Objectives:**

To develop a method for detecting jaw positions during jaw-tracking delivery to ensure an accurate delivery of radiation to patients using an electronic portal imaging device (EPID) in the cine mode on a linear accelerator for radiotherapy.

**Materials:**

A bidirectional picket fence (BPF) plan was used in a novel application to detect jaw positions during jaw-tracking delivery. In the BPF plan, jaws tracked multileaf collimator (MLC) apertures. The irradiated patterns were acquired by an EPID in the cine mode.

**Methods:**

The upper- and lower-half leaves in the MLC moved in opposite directions to facilitate detection of jaw positions on EPID images. A picket-fence-like image was created by summing all acquired cine images and evaluated to detect MLC leaf positions.

**Results:**

Jaw positions determined on the cine images were compared with those expected from the delivered BPF plan. The absolute differences (average ± 1 standard deviation) were 0.16 ± 0.19 mm for the X1 jaw and 0.11 ± 0.16 mm for the X2 jaw. The maximum error in the MLC leaf positions detected in the picket-fence-like pattern were 0.11 mm.

**Conclusions:**

Jaw positions during jaw-tracking delivery were identified using the cine EPID images and could be determined within an accuracy better than 0.5 mm. The BPF plan is also available as a picket fence test and can determine the MLC leaf positions to an accuracy better than 0.5 mm.

## Introduction

Radiation therapy (RT) using linear accelerators (linacs) can deliver volumetric modulated arc therapy (VMAT), which simultaneously allows delivery of a concentrated dose to a target and spare dosing to surrounding normal tissues^1)^. VMAT is a dynamic arc irradiation with the complex modulation of the gantry speed, the shape of irradiation fields formed by multileaf collimators (MLCs), and dose rate.

Linacs move collimator jaws located at the upstream of the MLC to block the interleaf transmission in VMAT, which is termed the *jaw-tracking technique* (JTT). The JTT can reduce doses delivered to normal tissues surrounding a target volume^2)^. Several researchers reported that compared with the conventional static jaw technique, use of the JTT can reduce the dose to normal tissues^3-5)^. To accurately deliver VMAT with JTT, jaws must move to the position defined in a treatment plan. If the jaw moves to an incorrect position, delivered dose distribution can differ from that calculated by the treatment planning system^6)^. Matsubayashi et al. reported that a dose error to normal tissue can be 0.179% when the jaw position error is 1.0 mm, which can be minimized by routinely controlling the accuracy of jaw positions^7)^. Faungrod et al. reported a method to detect jaw positions during VMAT with JTT using cine images acquired using an electronic portal imaging device (EPID) mounted on a linac^8)^. Their method is useful for patient- specific quality assurance (QA).

It is desirable to check jaw positions during JTT delivery as routine QA. In this work, we propose a new jaw position detection method for JTT by extending the conventional QA method for MLC and evaluate its accuracy for jaw position detection.

## Materials and Methods

### Overview

**Figure 1:**
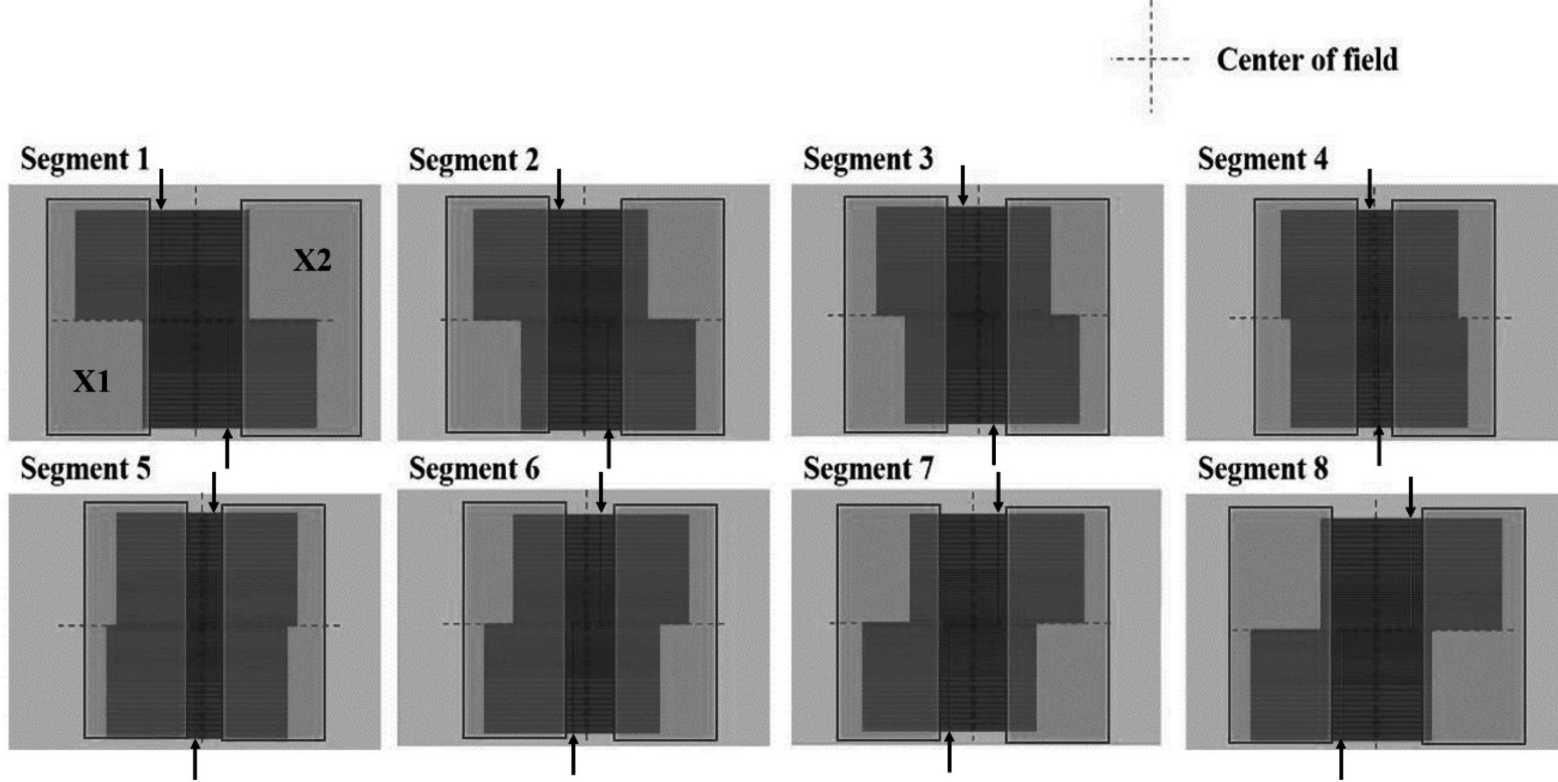
Multileaf collimator (MLC) jaw pattern of the bidirectional picket fence (BPF) plan showing the MLC leaves and jaws in each segment. The plan consists of eight MLC segments with an MLC aperture of 2.0 mm. The MLC-aperture centers of the upper and lower leaves in each segment are 70 mm, 50 mm, 30 mm, 10 mm, −10 mm, −30 mm, −50 mm, and −70 mm for the upper leaves and −70 mm, −50 mm, −30 mm, −10 mm, 10 mm, 30 mm, 50 mm, and 70 mm for the lower leaves, respectively. The left-sided jaw (X1) and right-sided jaw (X2) are set to be 5.0 mm retracted from the nearest MLC tips. Arrows indicates the positions of the MLC-aperture centers.

One of the most important aspects of quality control for use of MLCs is the accuracy of positioning. A common method to check MLC positions is the *picket fence* (PF) test^9, 10)^. In the PF test, the several slits with the MLC aperture of 1-2 mm are delivered repeatedly with separations of a few centimeters. In the conventional PF test, the jaw edges in the MLC transmission region cannot be detected on the EPID image because the dose from the MLC aperture is high. To resolve this problem, a new MLC pattern was created, termed the *bidirectional picket fence* (BPF) *pattern* as described in this study. Figure 1 shows the jaw and MLC positions in the BPF pattern. In the figure, the upper, lower, left, and right directions correspond to the Y2, Y1, X1, and X2 directions in the Varian IEC scale. In the BPF pattern, the upper leaves of MLC move in the left-to-right direction, and lower leaves move in the opposite direction. The left-sided jaw (X1) moves from the left to right with the edge following the aperture of the upper MLC leaves in segments 1, 2, 3, and 4 and moves in the opposite direction with the edge preceding the aperture of the lower MLC leaves in segments 5, 6, 7, and 8. The right-sided jaw (X2) moves from the right to left with the edge following the aperture of the lower MLC leaves and moves in the opposite direction with the edge preceding the aperture of the upper MLC leaves in segments 5, 6, 7, and 8 (Figure 1). In the bidirectional pattern, the jaw edge in the MLC transmission region can be seen without the disturbance from the MLC slit because a sufficient gap is maintained between the edge of the right-sided (left-sided) jaw and the upper (lower) MLC slit in segments 1, 2, 3, and 4 (Figure 1). For segments 5, 6, 7, and 8, a sufficient gap is maintained between the edge of the right-sided (left-sided) jaw and the lower (upper) MLC slit. We created the Digital Imaging and Communications in Medicine (DICOM) RT plan in which jaws were set 5.0 mm from the nearest MLC slit in the jaw- tracking mode. The BPF plan was imported into a linac (TrueBeam, Varian Medical Systems, Palo Alto, CA, USA) and cine images were acquired with aS 1200 EPID mounted on the linac. The cine images were analyzed to detect jaw positions as described in following text.

This article does not contain any studies with human participants performed by any of the authors.

### Bidirectional Picket Fence Plan

The Millennium 120 MLC, which has 120 leaves, was mounted on the linac. A dynamic MLC plan in which the upper leaves (leaf numbers 31-60) moving from the left to right and lower leaves (leaf numbers 1-30) moving from the right to left was created using MLC Shaper (Varian Medical Systems). Both upper leaves and lower leaves were stopped every 20 mm with a 2-mm aperture. The total number of the stopping points, or segments, was 8 (Figure 1). The MLC file was imported into Eclipse Ver. 13.6 (Varian Medical Systems) and exported in the DICOM RT Plan format. The exported plan was converted into a jaw-tracking BPF plan by defining jaw positions on each MLC segments. The jaw positions were 5.0 mm retracted from the nearest leaf edges (Figure 1). The plans were created as the static gantry mode at angles of 0 degrees, 90 degrees, and 270 degrees, and the VMAT mode with 360-degree full gantry rotation. The energy of X-ray was 6 MV, the dose rate was 600 MU/min, and the delivered MU was 120 MU. The jaw-tracking BPF plan was irradiated in a dynamic mode.

### Cine Image Acquisition with aS 1200 EPID

All measurements were performed using the TrueBeam linac. Cine images were acquired with aS 1200 EPID. An aS 1200 EPID was an amorphous silicon detector array and had an active area of 43 cm × 43 cm with 1280 × 1280 pixels and a resolution of 0.336 mm at a source to imager distance (SID) = 100 cm. All data were acquired in the cine imaging mode. The cine images were exported in the DICOM RT format from the Image Processing Service (IPS) in the TrueBeam service mode to verify the jaw positions.

### Jaw Detection with Image Processing

**Figure 2:**
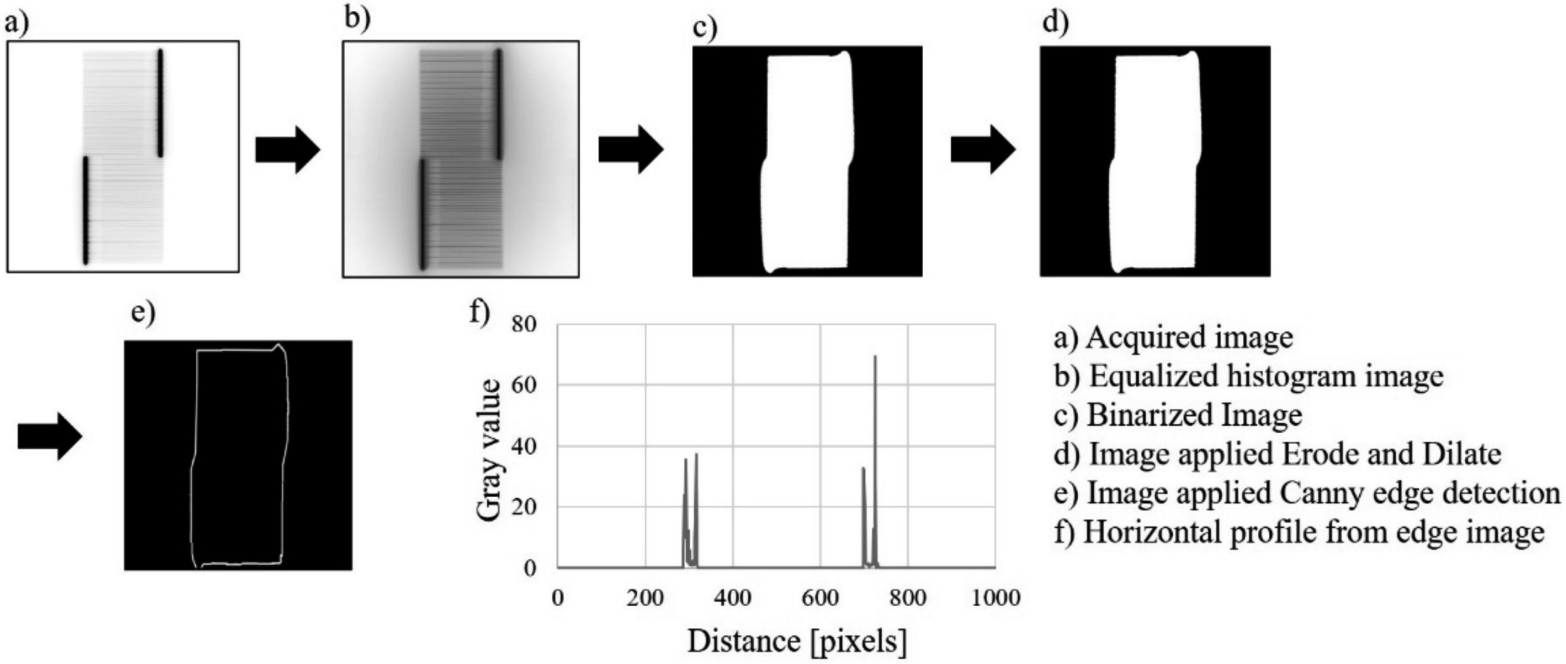
Workflow to detect jaw positions from cine images for segment 8. The one-dimensional horizontal profile (f) was obtained by summing up the binarized edge image (e) in the vertical direction. The inner two peaks correspond to the jaw edges and the outer two peaks correspond to the edges of the multileaf collimator (MLC) apertures.

Jaw positions were verified by analyzing the acquired cine images in the following workflow for each segment. The analyses were performed in an in-house script in an image processing package (Fiji, National Institutes of Health, Bethesda, MD, USA)^11)^. Figure 2 shows the workflow of the jaw detection method in this study. The signal of the jaw edge was much lower than the signal from the MLC-defined aperture because the jaw edge was always blocked by the MLC leaves in the BPF pattern (Figure 1). The cine images imported into Fiji were processed by the histogram equitation to enhance the image contrast (Figure 2b)^12-14)^. Based on the enhanced histogram, Otsu thresholding^15)^ was applied to binarize each image into a radiation-detected area and a radiation-blocked area (Figure 2b). After binarization, to remove small cavities and smooth any small and isolated areas, morphology processing (dilation and erosion)^16)^ was performed (Figure 2d). The Canny edge detection method^17)^ was used to extract the edges in the binarized image (Figure 2e). The binarized edge image was summed in the vertical direction to obtain a one-dimensional horizontal profile (Figure 2f). The profile had four peaks, with the inner two peaks corresponding to the jaw edges and the outer two corresponding to the edges of the MLC apertures. The peak positions of the inner two edges were recorded as the jaw positions. Several cine images were acquired for each segment. The image processing was applied to each acquired image and the mean and standard deviation (SD) of the jaw positions were calculated for each segment.

### Picket Fence Test

**Figure 3:**
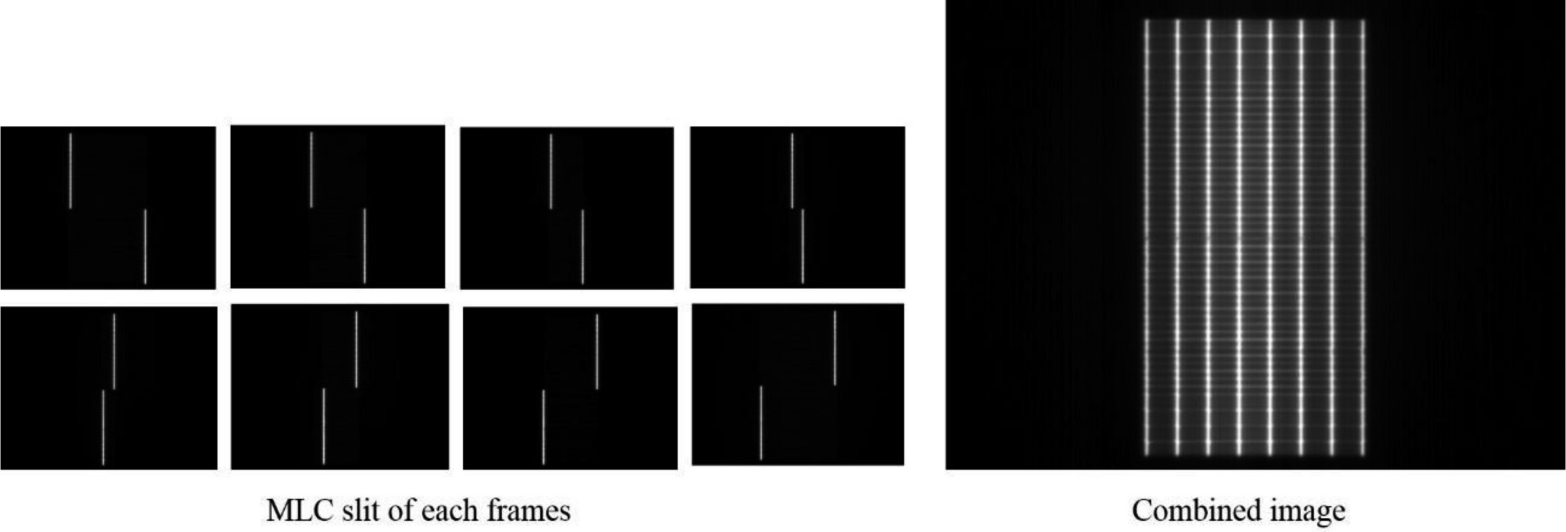
Creating a multileaf collimator (MLC) picket-fence-like image from all segment frames of bidirectional picket fence (BPF) plan.

The images obtained in all segments were accumulated to create a PF-like image to verify MLC leaf positions. Figure 3 shows the cine image in each segment (left) and the accumulated PF-like image (right). The PF-like images were analyzed with the picket fence module of Pylinac^18, 19)^. We evaluated the passing rate, the maximum error, and the average error of the position of MLC leaves in the PF-like image with 0.5-mm leaf tolerance value.

### Validation Jaw Position Accuracy with Static- Jaw and Error-Added BPF Plan

To verify the validity of our method of jaw detection, BPF plans with static jaw positions were created. The X1 and X2 jaw positions in this plan were −90.0 mm and +90.00 mm, respectively. Cine images were acquired with the static-jaw plans and analyzed using the same method as the jaw- tracking mode to detect jaw positions. Plans that had artificial errors on the jaw and leaf positions were also created to evaluate the accuracy of jaw position detection and sensitivity against positioning error of jaws and leaves. The artificial errors for the jaw and leaf position were 0.5 mm or 1.0 mm. The obtained cine images with the error-added plans were also analyzed in the same way as described above to detect the jaw and leaf positions.

## Results

### Detected Jaw Positions

#### Verification of detection accuracy of jaw position in the static-jaw BPF plan

Table 1 summarizes the jaw positions in the static jaw position BPF plan. The planned X1 and X2 jaw positions were −90.0 mm and +90.0 mm, respectively; the detected jaw positions at each segment (average ± 1 SD) of X1 and X2 were −90.06 ± 0.06 mm and +90.02 ± 0.13 mm, respectively. The detected jaw positions were in good agreement with the planned positions. This finding indicates that our method can accurately detect jaw positions under the MLC leaves (MLC transmission region) in the BPF plan.

**Table 1:** Summary of detected and planned jaw positions in static jaw plans

	X1 jaw position (mm)					X2 jaw position (mm)			
Segment	Planned	Detected	SD	Diff		Planned	Detected	SD	Diff
1	−90.00	−90.06	0.05	0.06		90.00	89.97	0.04	0.03
2	−90.00	−90.13	0.03	0.13		90.00	89.95	0.04	0.05
3	−90.00	−89.93	0.09	−0.07		90.00	90.03	0.04	−0.03
4	−90.00	−90.07	0.04	0.07		90.00	90.11	0.05	−0.11
5	−90.00	−90.06	0.22	0.06		90.00	90.26	0.04	−0.26
6	−90.00	−90.14	0.34	0.14		90.00	90.08	0.05	−0.08
7	−90.00	−90.05	0.14	0.05		90.00	89.89	0.05	0.11
8	−90.00	−90.03	0.05	0.03		90.00	89.89	0.06	0.11

Diff: Planned-Detected; SD: Standard deviation.

#### Detected jaw positions in the jaw-tracking BPF plans

The detected jaw positions in the jaw-tracking BPF plans with gantry angles of 0, 90, and 270 degrees, and 360-degree rotation are shown in Table 2 (X1) and Table 3 (X2). For the X1 (X2) jaw, the absolute differences between detected and expected jaw positions (average ± 1 SD) were 0.12 ± 0.17 mm (0.12 ± 0.15), 0.18 ± 0.19 mm (0.12 ± 0.17), 0.10 ± 0.21 mm (0.06 ± 0.11), and 0.16 ± 0.21 mm (0.14 ± 0.22) with the gantry angles of 0, 90, and 270 degrees, and 360-degree rotation, respectively.

**Table 2:** Planned and detected jaw positions of X1 jaw (mm)

		Gantry 0 degrees				Gantry 90 degrees				Gantry 270 degrees				Gantry rotation		
Segment	Planned	Detected	SD	Diff		Detected	SD	Diff		Detected	SD	Diff		Detected	SD	Diff
1	−74.00	−73.78	0.24	−0.22		−73.92	0.10	−0.08		−73.99	0.24	−0.01		−73.71	0.33	−0.29
2	−54.00	−54.06	0.12	0.06		−54.10	0.13	0.10		−54.13	0.17	0.13		−54.10	0.1	0.10
3	−34.00	−33.81	0.21	−0.19		−33.69	0.24	−0.31		−34.11	0.17	0.11		−34.27	0.13	0.27
4	−14.00	−13.81	0.27	−0.19		−13.99	0.19	−0.01		−13.96	0.22	−0.04		−13.83	0.13	−0.17
5	−16.00	−16.06	0.05	0.06		−15.73	0.29	−0.27		−16.14	0.16	0.14		−15.91	0.13	−0.09
6	−36.00	−35.93	0.17	−0.07		−36.18	0.23	0.18		−36.29	0.19	0.29		−36.19	0.37	0.19
7	−56.00	−56.13	0.19	0.13		−55.70	0.28	−0.30		−55.99	0.3	−0.01		−56.04	0.14	0.04
8	−76.00	−76.02	0.12	0.02		−76.18	0.03	0.18		−76.05	0.19	0.05		−75.87	0.36	−0.13

Diff: Planned-Detected; SD: Standard deviation; X1: left-sided jaw.

**Table 3:** Planned and detected jaw positions of X2 jaw (mm)

		Gantry 0 degrees				Gantry 90 degrees				Gantry 270 degrees				Gantry rotation		
Segment	Planned	Detected	SD	Diff		Detected	SD	Diff		Detected	SD	Diff		Detected	SD	Diff
1	76.00	76.23	0.20	−0.23		76.03	0.07	−0.03		76.04	0.01	−0.04		75.72	0.24	0.28
2	56.00	55.89	0.23	0.11		55.87	0.07	0.13		55.99	0.04	0.01		56.09	0.45	−0.09
3	36.00	35.92	0.15	0.08		36.08	0.22	−0.08		36.19	0.1	−0.19		35.83	0.23	0.17
4	16.00	15.85	0.32	0.15		15.78	0.21	0.22		15.98	0.17	0.02		15.88	0.02	0.12
5	14.00	14.25	0.09	−0.25		14.09	0.17	−0.09		14.01	0.12	−0.01		13.77	0.24	0.23
6	34.00	34.00	0.02	0.00		33.83	0.23	0.17		34.08	0.11	−0.08		33.90	0.2	0.10
7	54.00	54.02	0.17	−0.02		53.86	0.25	0.14		53.98	0.14	0.02		53.96	0.19	0.04
8	74.00	74.09	0.04	−0.09		73.90	0.14	0.10		73.93	0.16	0.07		73.89	0.19	0.11

Diff: Planned-Detected; SD: Standard deviation; X2: right-sided jaw.

#### Detected jaw positions in the jaw-tracking BPF plans with artificial errors

Table 4 shows the detected jaw positions in the BPF plans with artificial errors of 0.5 mm or 1.0 mm. The absolute differences between detected and expected jaw positions including artificial errors (average ± 1 SD) were 0.14 ± 0.10 mm for the X1 jaw and 0.13 ± 0.15 mm for the X2 jaw. This result shows that our method can detect a jaw positioning error of 0.5 mm with an accuracy of 0.3 mm.

**Table 4:** Detected and planned jaw positions with artificial errors at gantry 0 degrees

	X1 jaw position (mm)					X2 jaw position (mm)			
Segment No.	Planned	Detected	SD	Diff		Planned	Detected	SD	Diff
1	−73.00	−73.19	0.16	0.19		75.00	75.12	0.19	−0.12
2	−53.50	−53.56	0.02	0.06		56.50	56.39	0.11	0.11
3	−34.50	−34.55	0.13	0.05		37.00	36.62	0.17	0.38
4	−15.00	−15.26	0.05	0.26		15.50	15.37	0.15	0.13
5	−15.00	−15.22	0.15	0.22		15.00	14.83	0.27	0.17
6	−35.50	−35.74	0.14	0.24		33.50	33.52	0.08	−0.02
7	−57.00	−57.06	0.15	0.06		53.00	52.94	0.05	0.06
8	−76.50	−76.51	0.08	0.01		74.50	74.48	0.10	0.02

Diff: Planned-Detected; SD: Standard deviation; X1: left-sided jaw; X2: right-sided jaw.

### Picket Fence Test Using Accumulated Cine Images

**Figure 4:**
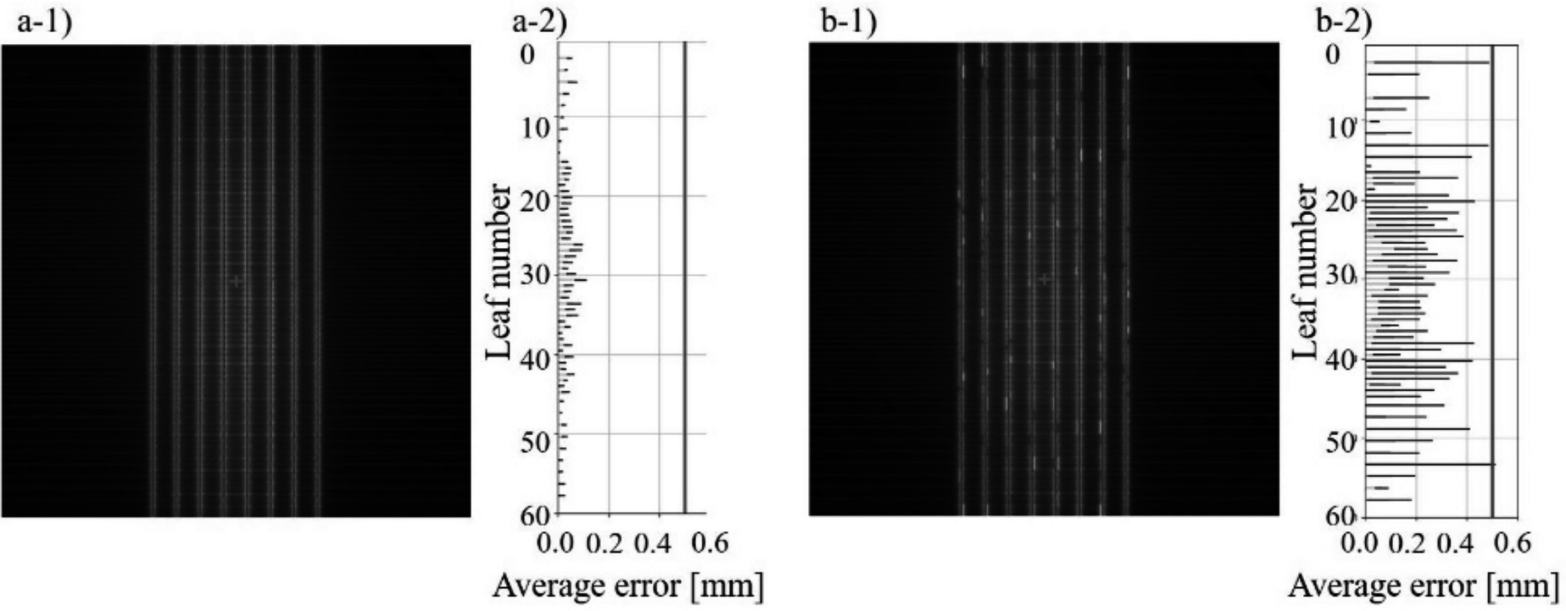
Analysis of the multileaf collimator (MLC) picket fence (PF) test with Pylinac PF module. a-1) PF test without artificial error; a-2) error histogram of PF test without artificial error; b-1) PF test with artificial error; b-2) error histogram of PF test with artificial error. The vertical line in each error histogram shows a 0.5 mm leaf tolerance value.

Figure 4 shows the results of analysis for the PF-like images using Pylinac. Table 5 shows the passing rates with a leaf tolerance value of 0.5 mm and the maximum error and average error of leaf positions. All the leaf positions were within a tolerance of 0.5 mm at all gantry angles. The maximum error was 0.11 mm in the BPF plan with a gantry rotation of 360 degrees. In the plan with the artificial error for MLC leaf positions, the passing rate was 95.2 % and the maximum error was 0.97 mm. The maximum artificially added leaf position error was 1.0 mm. Consequently, use of the PF-like image from the BPF plan can accurately detect leaf positioning errors. The PF test, as MLC QA, can be performed with the BPF plan.

**Table 5:** Results of the picket fence test from Pylinac with BPF plan

	Gantry angle			
	0 degrees	90 degrees	270 degrees	360-degrees rotation
Leaves passing (%)	100.00	100.00	100.00	100.00
Absolute median error (mm)	0.01	0.03	0.02	0.02
Maximum error (mm)	0.06	0.09	0.05	0.11

## Discussion

The JTT is effective to reduce the dose from radiation leakage transmitted by MLCs. The transmitted dose can reach 1.5% of the primary dose^20)^. The uncertainty of jaw positioning affects the dose distribution delivered to a patient. In a study that evaluated the impact of jaw positioning uncertainty in conformal plans, a jaw positioning error of 10 mm can lead to a 5.0% dose error^21)^. To date, few studies have investigated the influence of jaw positioning uncertainty in VMAT with JTT, although some studies reported that jaw positioning uncertainty affects dose distribution^6, 7)^. Therefore, it is important in high-precision RT to control the accuracy of jaw position. In this study, we developed and evaluated a method to verify jaw positions in JTT delivery with a novel MLC motion pattern─the BPF pattern. This method uses an EPID equipped with a linac and freely available image processing software. Specific devices are not otherwise needed. Furthermore, the MLC motion pattern of the BPF is similar to a conventional MLC QA pattern, termed the *picket fence pattern*. A PF-like image can easily be created from the acquired images using the BPF pattern and analyzed using the conventional MLC QA method. Therefore, the verification of jaw positions in JTT and MLC positions can be performed simultaneously. Our proposed method allows verification with low cost and low workload, is quick, and provides accurate jaw position QA in jaw-tracking delivery.

**Figure 5:**
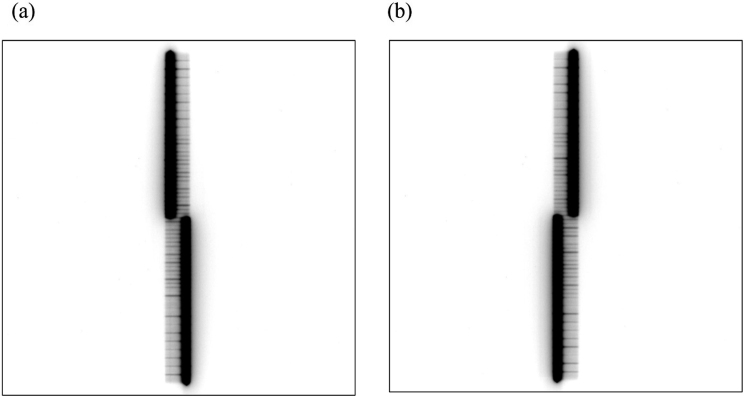
Cine image frames in segment 4 and 5: a) cine image frame of segment 4; b) cine image frame of segment 5.

Detected jaw positions in the BPF plan with a static gantry were in good agreement with the planned positions. The absolute differences between the planned and detected positions were 0.16 ± 0.19 mm for the X1 jaw and 0.11 ± 0.16 mm for the X2 jaw. This finding suggests that jaw position can be detected even in a region where only transmitted radiation exists and a jaw image is difficult to see because of the radiation from the MLC-defined aperture. In particular, the region of MLC transmission is smallest (23 mm) in the segment numbers 4 and 5 because the jaw aperture here is smallest among all segments (Figure 5). Even in such a situation, our method can detect jaw positions because Otsu thresholding works effectively to enhance contrast.

The average of the absolute differences between the detected and planned jaw positions in the BPF pattern with the artificial errors were 0.14 ± 0.10 mm (average ± 1 SD) for the X1 jaw and 0.13 ± 0.15 mm for the X2 jaw. The maximum absolute differences were 0.26 mm for the X1 jaw and 0.38 mm for the X2 jaw. The gantry angle dependence was not observed.

From Tables 1 and 2, we estimated the jaw position can be detected with a precision within 0.5 mm, because the maximum absolute difference was 0.31 mm (X1, segment 3, gantry angle 90 degrees). The report of American Association of Physicists in Medicine Task Group 142 recommends that the positioning accuracy of an asymmetric jaw should be within 1.0 mm^22)^. Our method can satisfy the criterion, because the maximum absolute average ± 1 SD was 0.62 mm (X1, segment 1, gantry rotation).

A similar method to simultaneously detect jaw and MLC positions is the machine performance check (MPC) method, which is one of the major modes of TrueBeam. In MPC, the MLC position is detected with a comb-like MLC pattern and the jaw position is detected in an 18 × 18 cm^2^ open field. A study that compared MPC with external QA procedures reported that X jaw offsets were detected within 0.10 mm^23)^. However, the MPC method evaluates only a single static jaw position, whereas our method can evaluate jaw positions in eight different segments and multiple MLC positions, equivalent to the capabilities of the conventional PF pattern.

The limitation of our method is that Y jaw positions with JTT cannot be evaluated in the present BPF pattern. Although static Y jaw positions can be evaluated with the same method as X jaws, a PF-like image cannot be created when JTT is applied to Y jaws. We are currently developing a pattern that can create a PF-like image even if JTT is applied to the Y jaws, and we will report our study results in a future publication.

In conclusion, we developed the BPF plan to verify jaw positions during jaw-tracking delivery. Using the BPF plan, the positions of jaws were verified with accuracy of 0.16 ± 0.19 mm for the X1 jaw and 0.11 ± 0.16 mm for the X2 jaw. Furthermore, we verified that a PF-like image can be created by accumulating the cine images and used as a conventional PF test for MLC.

## Funding

No funding was received.

## Author contributions

TK, SS, CK, KU, TI, HN, HW, and KS participated in the study design and data interpretation. KT and SS performed the measurements. KT mainly analyzed and interpreted the measured data. KT and SS were major contributors in writing the manuscript. All authors read and approved the final manuscript.

## Conflicts of interest statement

The authors declare that there are no conflicts of interest.
